# Comparing the Characteristics of Amniotic Membrane-, Endometrium-, and Urinary-Derived ECMs and Their Effects on Endometrial Regeneration in a Rat Uterine Injury Model

**DOI:** 10.3389/fbioe.2022.861496

**Published:** 2022-04-13

**Authors:** Wanqing Ji, Jiaming Wen, Weige Lin, Ping He, Bo Hou, Song Quan

**Affiliations:** ^1^ Department of Gynecology and Obstetrics, NanFang Hospital, Southern Medical University, Guangzhou, China; ^2^ Guangzhou Women and Children’s Medical Center, Guangzhou Medical University, Guangzhou, China; ^3^ Guangdong Maoming Health Vocational College, Maoming, China; ^4^ Department of Neurosurgery, Third Affiliated Hospital of Sun Yat-sen University, Guangzhou, China

**Keywords:** uterine injury, decellularized extracellular matrix, amniotic membrane, urethra, endometrial regeneration, inflammatory response

## Abstract

The decellularized extracellular matrices (d-ECMs) currently utilized to repair endometrial injuries are derived from three tissue sources, the endometrium (dE-ECM), placental amniotic membrane (dA-ECM), and urinary (dU-ECM). Notably, the structures of dU-ECM and dE-ECM are similar. These d-ECMs are derived from different tissues, and their specific roles in endometrial injury repair remain unclear. This study aimed to analyse the characteristics of the tissue microstructures and compositions to confirm specific differences among the three ECM types. And using a rat model of endometrial injury, the effects of all the matrices after implantation *in vivo* on the promotion of endometrial regeneration were analysed. After decellularization, dE-ECM had more residual active factors than the other two ECM types, while dA-ECM had significantly less DNA, α-Gal antigen components and extracellular matrix components than the other two groups. Although the three ECMs had no effect on the proliferation of stromal cells *in vitro*, dA-ECM may have increased the sensitivity of stromal cells to oestradiol (E2) responses. *In vivo* experiments confirmed the promotional effect of dA-ECM on endometrial regeneration. For example, the endometrial thickness, collagen deposition, endometrial tissue regeneration, vascular regeneration and pregnancy outcomes were significantly better in this group than in the other two groups. These findings might be associated with the excellent immune tolerance of dA-ECM. Therefore, when selecting a d-ECM for the treatment of endometrial injury, dE-ECM, which has the strongest tissue specificity, is not the preferred choice. Controlling the inflammatory responses in local lesions at the early stage may be a prerequisite for ECMs to exert their functions.

## Graphical Abstract

Overall experimental design scheme.
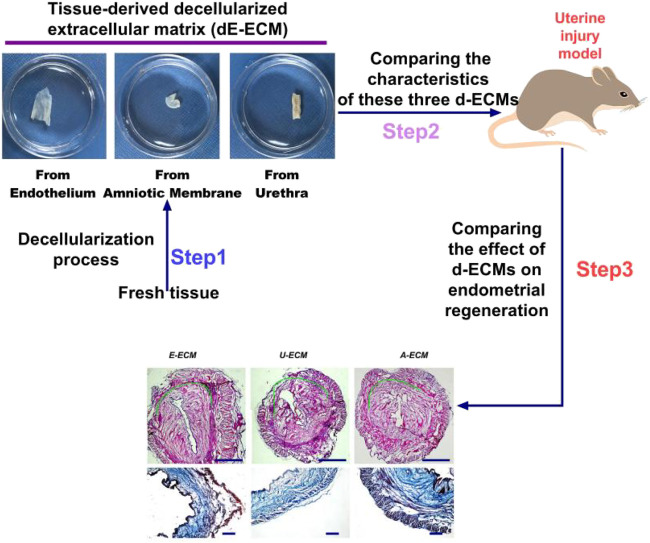

## 1 Introduction

Intrauterine adhesion (IUA) is a disease characterized by partial or complete uterine/cervical atresia (Yu et al., 2008). Endometrial stem cells (ESCs) are the most critical factors for endometrial regeneration (Murakami et al., 2014). Under normal physiological conditions, ESCs in their biological niche, the endothelium of the spiral arterioles in the basal layer, provide support to both the epithelial and stromal compartments and generate intrinsic stem or progenitor cells every menstrual cycle (Xu et al., 2020). In clinical practice, injury or inflammation damages the basal layer of the endometrium. Eventually, hormone-responsive endometrial tissues are replaced by excessively deposited avascular and nonfunctional fibrous tissues, accompanied by a decrease in or disappearance of glands. These features of IUA seriously affect the fertility of women of childbearing age.

Advances in tissue engineering have yielded biomaterials that play important roles in repairing damaged tissues and provide a method for repairing the damaged basal endometrial layer, which causes reproductive dysfunction (Li et al., 2021). The extracellular matrix (ECM) is a dynamic and complex environment with specific biophysical and biochemical properties (Liu et al., 2021). In the endometrial microenvironment, the ECM comprises a complex mixture of glycosaminoglycans (GAGs), proteoglycans, and fibrous structural proteins that interact with the resident cells and play important roles in cell growth, development, homeostasis, regulation, and maturation (Cook et al., 2017). The decellularized ECM (d-ECM) is isolated from original fresh tissues and has become a promising natural biomaterial that is often used in supporting, replacing, and regenerating damaged tissues. Decellularization is achieved via exposure of the ECM (Zhang et al., 2022), and d-ECM can be easily collected from various tissues/organs by selecting an appropriate decellularization method. The d-ECM can mimic the native environment under *in vitro* conditions (e.g., by creating a biomimetic environment allowing for the growth of stromal cells). Recent studies have confirmed that biomaterials with tissue-specific ECM features can improve regeneration and support gestation in a severe uterine damage model (Miyazaki and Maruyama, 2014; Campo et al., 2021), thus demonstrating the value of decellularized tissues (Ma et al., 2021).

When selecting appropriate d-ECM materials, tissue-specific ECMs should be considered (Miki et al., 2019; Li et al., 2021), as they are thought to better aid in restoring the functional characteristics of cells. Some researchers have proposed that, relative to other d-ECMs, a biologically native ECM derived from decellularized endometrial tissues renders a more physiologically suitable microenvironment and creates a more biomimetic environment to allow the growth of stromal cells (Campo et al., 2019). Several studies have already confirmed that tissue-specific ECM has beneficial effects in guiding the differentiation of stem cells and preserving the phenotypes of primary cultured cells (Chen et al., 2019a; Abazari et al., 2020). Therefore, the current choices of d-ECM for repairing endometrial injury are mainly concentrated in three tissue origins: endometrial tissue-derived decellularized extracellular matrix (dE-ECM), which has the strongest specificity (Campo et al., 2019); amniotic membrane (AM)-derived decellularized extracellular matrix (dA-ECM) from the foetal placenta (Chen et al., 2021), which is used most extensively; and urinary tissue-derived decellularized extracellular matrix (dU-ECM), which has a structure similar to that of uterine dE-ECM (Zhang et al., 2020). All three of these ECMs have certain promotional effects on the repair of endometrial injury.

Notably, the experimental conditions and models as well as the research purposes of the previous relevant studies differ, and the structures and protein compositions of these three d-ECM types are obviously different. Therefore, their unique roles in endometrial repair have not yet been verified.

This study compared the characteristics of these three d-ECMs and their therapeutic effects on endometrial repair under the same conditions, aiming to further clarify their specific promotional effects on endometrial injury repair and to explore the mechanism underlying endometrial repair from the aspect of the ECM, thus providing new ideas and methods for designing engineering biomaterials for endometrial injury repair.

## 2 Methods and Materials

### 2.1 Animals and Ethics

In this study, eight-week-old healthy female Sprague-Dawley (SD) rats (weighing 200–230 g) were used for the *in vivo* experiments. Fresh endometrial tissues and urinary tract specimens were obtained from SD rats, and AM tissues were collected from SD rats with a normal pregnancy (E15, used for other experiments). All animals were provided by the Animal Experiment Center of Sun Yat-sen University. Next, rat vaginal secretion smears were observed every day, and all surgeries were performed during the diestrus period. The experimental programme was approved by the Experimental Animal Management and Ethics Committee of Sun Yat-sen University. Endometrial stromal cells were purchased from Procell (China).

### 2.2 d-ECM Preparations

#### 2.2.1 dA-ECM

Placentas were acquired under aseptic conditions. The AM was isolated from villus tissues using blunt dissection and washed thoroughly with 0.9% sodium chloride (NaCl) three times to remove residual blood. AM tissues were cut into 2 × 2 mm tissues blocks. Fresh AM tissues were incubated with peracetic acid (2 M, pH 2.5) for 20 min at room temperature, washed with phosphate-buffered saline (PBS) three times (10 min/wash), decellularized using 2% (w/v) sodium dodecyl sulfate (SDS) (Sigma-Aldrich, USA) solution for 2 h at room temperature, and washed with PBS three times (10 min/wash).

#### 2.2.2 dE-ECM

Fresh endometrial tissues were collected and washed with PBS (0.1 M, pH 7.4; Gibco, USA). The dE-ECM was prepared as previously described (Campo et al., 2019). Endometrial tissues were washed with PBS for 1 h. The tissues were then (1) decellularized using 0.1% SDS on a shaker for 18 h, (2) washed with distilled water for 30 min, (3) treated with 1% Triton X-100 (Sigma-Aldrich, USA) for 30 min, and (4) washed with PBS for 4 h. The above Steps 1–4 were repeated for two cycles. All the above procedures were performed at room temperature on a shaker (150 rpm/min).

#### 2.2.3 dU-ECM

Urinary bladder mucosa (UBM) derived from the rat urinary tract was prepared as follows: the tissue was washed with PBS, incubated in PBS containing 1.28 M NaCl for 1 h, incubated in TE buffer containing 1% SDS for 24 h, and washed with deionized water for 24 h. The deionized water was replaced every 12 h.

The above decellularization scaffolds were sterilized with 0.1% peracetic acid (PAA) and stored for subsequent use (at 4°C).

### 2.3 d-ECMs Characterization

#### 2.3.1 Haematoxylin-Eosin Staining

The dA-ECM, dE-ECM, and dU-ECM samples were fixed with 4% paraformaldehyde for 6 h, and frozen sections (5–10 μm) were thereafter prepared. Staining was performed with an HE reagent kit (Leagene, China) in accordance with the manufacturer’s instructions, and the samples were observed under an upright microscope. In each group, 10 random fields were photographed and recorded at high magnification (×200).

#### 2.3.2 Scanning Electron Microscopy

For SEM analysis, the samples were fixed with 10% glutaraldehyde for 6 h, postfixed with 4% osmium tetroxide for 4 h, dehydrated in a graded ethanol series, soaked in isopentyl acetate for 15 min to replace the ethanol, and dried at the critical point for 6 h (Hitachi, Tokyo, Japan). The dried specimens were coated with gold and observed under a Quanta 200 SEM instrument (FEI, USA).

#### 2.3.3 DNA Quantitative Analysis

Quantitative analysis of the DNA content was conducted as follows: DNA was isolated from the samples using a Fast DNA Tissue Kit (QIAGEN, Hilden, Germany) and quantified using PicoGreen (Invitrogen, Waltham, MA, United States).

#### 2.3.4 α-Gal ELISA

The test specimens were washed thoroughly with PBS (4°C, 0.02 M, pH 7.0–7.2), cut into small pieces, and homogenized with a glass tissue homogenizer. The cell membranes were further disrupted by sonication. The samples were then centrifuged (1,500×g, 15 min), and the supernatants were collected for further analyses. An α-galactosidase ELISA kit (BlueGene Bio-Connect, Huissen, Netherlands) was used to quantify the α-Gal content. The optical density (OD) was immediately read at 450 nm using a microtiter plate reader.

### 2.4 Quantitation of GAG and Collagen Contents

A collagen detection reagent kit (Biocolor, County Antrim, United Kingdom) was used to measure the collagen content in the samples. Briefly, the samples were solubilized in acetic acid (0.5 M) supplemented with pepsin (1 mg/ml (w/v); Sigma-Aldrich, USA) overnight in a refrigerator to dissolve the collagen extract in acid/pepsin. Next, the hydrolysates were treated with Sircol dye reagent (1 ml) for 30 min at room temperature. The absorbance of hydroxyproline in each sample was measured at 555 nm.

To analyse sulfated GAGs in the acid-hydrolysed experimental groups, a Blyscan assay kit (Biocolor, County Antrim, United Kingdom) was used according to the manufacturer’s instructions.

### 2.5 Detection of Residual Active Factors in the ECMs

The dA-ECM, dE-ECM, and dU-ECM samples were cut into small pieces, thoroughly lysed in radioimmunoprecipitation assay (RIPA) lysis buffer, and centrifuged at 10,000–14,000 g for 3–5 min. The supernatants were collected, and the levels of epidermal growth factor (EGF), fibroblast growth factor (FGF), and hepatocyte growth factor (HGF) were measured by ELISAs (R&D, USA). All operations were performed strictly in accordance with the manufacturers’ instructions.

### 2.6 Effects of the ECMs on the Proliferation and Secretion Functions of Stromal Cells

Lyophilized and pulverized dA-ECM, dE-ECM, and dU-ECM samples were placed in a 0.01 N HCL solution containing 1 mg/ml pepsin (Sigma) at a concentration of 10 mg ECM/mL and stirred at room temperature for 48 h. After each ECM was completely digested and an ECM solution formed (pH adjusted to 7.4 using 0.01 N NaOH), 1 ml of the ECM solution was incubated in a 48-well plate at 37°C overnight. Next, a suspension of 1 × 10^5^ stromal cells was added into each well and cultured at 37°C for 48 h. Cell Counting Kit-8 (CCK-8; Keygentec, China) solution was added to the culture medium at a ratio of 1:10 and incubated with the cells at 37°C in the dark for 2 h. Next, 100 μl of culture medium was added to a 96-well plate, and the OD at 450 nm was measured using a microplate reader. The cells were then labelled with the thymidine analogue 5-ethynyl-2-deoxyuridine (Edu) using an Edu reagent kit (Beyotime, China), and cell proliferation was determined by calculating the OD and differences in Edu expression.

Culture medium containing 10 pg/ml oestradiol (E2; Sigma-Aldrich, USA) was added to the each of the abovementioned groups, and the cells were cultured for 48 h. The cells were collected, and the messenger RNA (mRNA) expression levels of vascular endothelial growth factor (VEGF) and insulin-like growth factor (IGF)-1 were determined by reverse transcription-polymerase chain reaction (RT-PCR) to analyse the effects of the different ECM culture systems on the secretion functions of endometrial stromal cells after E2 stimulation.

Total RNA was extracted from cultured endometrial cells using an RNA sample kit (TIANGEN, China) and then reverse transcribed into complementary DNA (cDNA) using a PrimeScript reverse transcription (RT) kit (Takara). The primer sequences used to amplify each target gene were synthesized by Generay (Shanghai, China) (Table 1). The qRT-PCR reaction was carried out using a SYBR Premix Ex Tag^TM^ kit (Takara, Japan) on an ABI 7500 Thermocycler (Applied Biosystems, USA).

**TABLE 1:** Primers of specific genes used in qRT-PCR analyses.

Gene	Forward (5′-3′)	Reverse (5′-3′)
VEGF	TGT​TTC​CTG​TCC​ACG​CAA​TG	TGG​GCT​AAG​AGG​AAC​GCA​GA
IGF-1	TCA​GCA​GTC​TTC​CAA​CCC​AA	AAGGCGAGCAAGCACAGG
TGF-β	GCT​GGA​GAA​GCA​GAG​CGT​CT	CAC​ACC​CCA​CAG​AAC​TTA​GC
TIMP	CGC​TAG​AGC​AGA​TAC​CAC​GA	AGC​GTC​GAA​TCC​TTT​GAG​CA
PDGRBB	TGA​CCA​CTC​CAT​CCG​CTC​CT	CCA​GAA​TGT​GCT​CGG​GTC​AT
COLIA1	CCC​TGA​AGT​CAG​CTG​CAT​A	GGC​AGA​AAG​CAC​AGC​ACT​C
GAPDH	TAC​CCA​CGG​CAA​GTT​CAA​CG	CAC​CAG​CAT​CAC​CCC​ATT​TG

### 2.7 Animal Models

To construct the IUA model, rats were anaesthetized with an intraperitoneal injection of 10% chloral hydrate (0.3 ml/100 g; Sigma-Aldrich, USA). The abdominal cavity was opened under aseptic conditions to expose the bilateral uterine horns. The left side served as the experimental site, and the right side was used as a control. The uterus was dissected longitudinally at the left uterine horn (approximately 1 cm from the ovary), and the incision was approximately 2 cm long. Next, one-third of the endometrium on the lateral side was curetted with a surgical blade along the long axis, and uterine curettage was stopped when the pale endometrium was visible with the naked eye. The uterine and abdominal cavities were washed with sterile normal saline, and the uterus (6-0 absorbable suture) and abdominal wall skin (4-0 absorbable suture) were sequentially sutured. After surgery, the animals were given antibiotics for 3 d (intramuscular injection, 100,000 units penicillin for each rat).

A total of 180 healthy female rats were randomly divided into five groups. After establishment of the IUA model, the animals in each group were treated as follows: sham group (skin was cut to expose the uterus without damaging the uterus or endometrium), dA-ECM group (implantation of a dA-ECM scaffold that matched the damage range), dU-ECM group (implantation of a dA-ECM scaffold that matched the damage range), dE-ECM group (implantation of a dE-ECM scaffold that matched the damage range), and injury group (no scaffold implantation in the lesion area after injury and treatment with only normal saline).

### 2.8 Inflammatory Response

At 2 and 7 days after the operation, intracardiac blood was collected under general anaesthesia. Blood samples were placed in sterile Eppendorf (EP) tubes, allowed to clot at 4°C for 30 min, and then centrifuged (3,000 rpm/min) for 10 min. The centrifuged samples were used for ELISA detection (R&D Systems, USA). The serum levels of inflammatory cytokines, tumour necrosis factor (TNF) and b-FGF at the early and late stages were measured with a microplate reader at OD 450 nm to reflect the systemic inflammatory conditions of the animals of each group. A macrophage-specific marker (CD68; Cell Signaling Technology, USA) was used to evaluate inflammatory cell infiltration into local lesions, reflecting the local inflammatory conditions.

### 2.9 Profibrotic Cytokines

At 2 and 4 weeks after the operation, endometrial tissues were collected from lesioned areas, and the mRNA expression levels of profibrotic cytokines, tissue inhibitors of matrix metalloproteinase (TIMPs), platelet-derived growth factor-BB (PDGF-BB), transforming growth factor-β (TGF-β), and collagen type I α1 (COLIA1) were detected using RT-PCR (for the specific protocol, please refer to Section 2.6) to analyse the levels of endometrial fibrosis among all the groups.

### 2.10 Determination of Endometrial Structure Regeneration

Animals were euthanized at postoperative week 2 and 4 (12 rats in each group at each time point), after which the uterine horns were isolated, fixed in 4% paraformaldehyde for 12 h, and sliced into frozen sections (6 μm).

Staining was performed in accordance with the instructions provided with the HE reagent kit (Leagene, China) and Picro Sirius reagent kit (Leagene, China). The samples were observed under a microscope, and 10 surgical fields of each group were randomly selected, photographed, and recorded under high magnification (×200). Images were acquired using an Eclipse 80i microscope (Nikon, Japan) equipped with a high-resolution digital colour camera (Digital Sight US-U2, Nikon). The endometrial thickness was measured based on the HE staining results, and collagen deposition in the endometrium was determined based on the Picro Sirius staining results (deep red areas).

Immunofluorescence staining was performed as follows. Frozen tissue sections were sliced at a thickness of 5–10 μm, washed with PBS, incubated with a blocking solution containing 10% goat serum (Beyotime, China) and 0.3% Triton-x 100 (Sigma-Aldrich, USA) for 2 h, and then incubated with a primary antibody targeting Cytokeratin-18 (Abcam), Vimentin (Cell Signaling Technology), or CD31 (Abcam) at 4°C overnight. The sections were then removed from the refrigerator, warmed, washed with PBS (5 min each time for a total of three times), incubated with appropriate immunofluorescent secondary antibodies (Alexa Fluor® 488 or Alexa Fluor® 594, (both from Jackson ImmunoResearch) at room temperature for 60 min, and washed with PBS three times. Then, nuclei were counterstained with 25 μg/ml DAPI (Sigma-Aldrich, St. Louis, MO). Immunofluorescence staining images were acquired with a laser scanning microscope (LSM880 with Fast Airyscan, ZEISS), and the results were analysed with Image-Pro Plus software (Media Cybernetic, Inc.). CD31 is a reliable marker of vascular endothelial cells and can reflect the number of blood vessels in local endometrial tissues. Ki-67 is a classic cell proliferation marker, and CK18 and Vimentin are specific markers of endometrial glandular epithelial cells and stromal cells and are used to detect regeneration of the endometrial glandular epithelium and the lamina propria.

### 2.11 Pregnancy Testing

To determine whether the regenerated endometrium had the ability to support fertilized egg implantation and maintain normal pregnancy, we pre-mated female rats in each group with male Sprague-Dawley rats. Each group of animals underwent at least two estrous cycles prior to fertilization. Pregnancy was indicated by the presence of a vaginal plug. Experimental rats (*n* = 15 in each group) were euthanized by cervical dislocation on day 14 after conception, and the uterus was examined for embryos at the site of injury.

### 2.12 Statistical Analysis

Data are presented as the mean ± standard deviation (SD) and were analysed using GraphPad Prism 8.0 software (La Jolla, CA, United States) and Statistics Package for Social Science (SPSS 17.0). Statistical significance was determined by one-way analysis of variance (ANOVA) followed by the Tukey-Kramer post hoc test. *p* values < 0.05 were considered statistically significant.

## 3 Results

### 3.1 Morphological Evaluation

**FIGURE 1:**
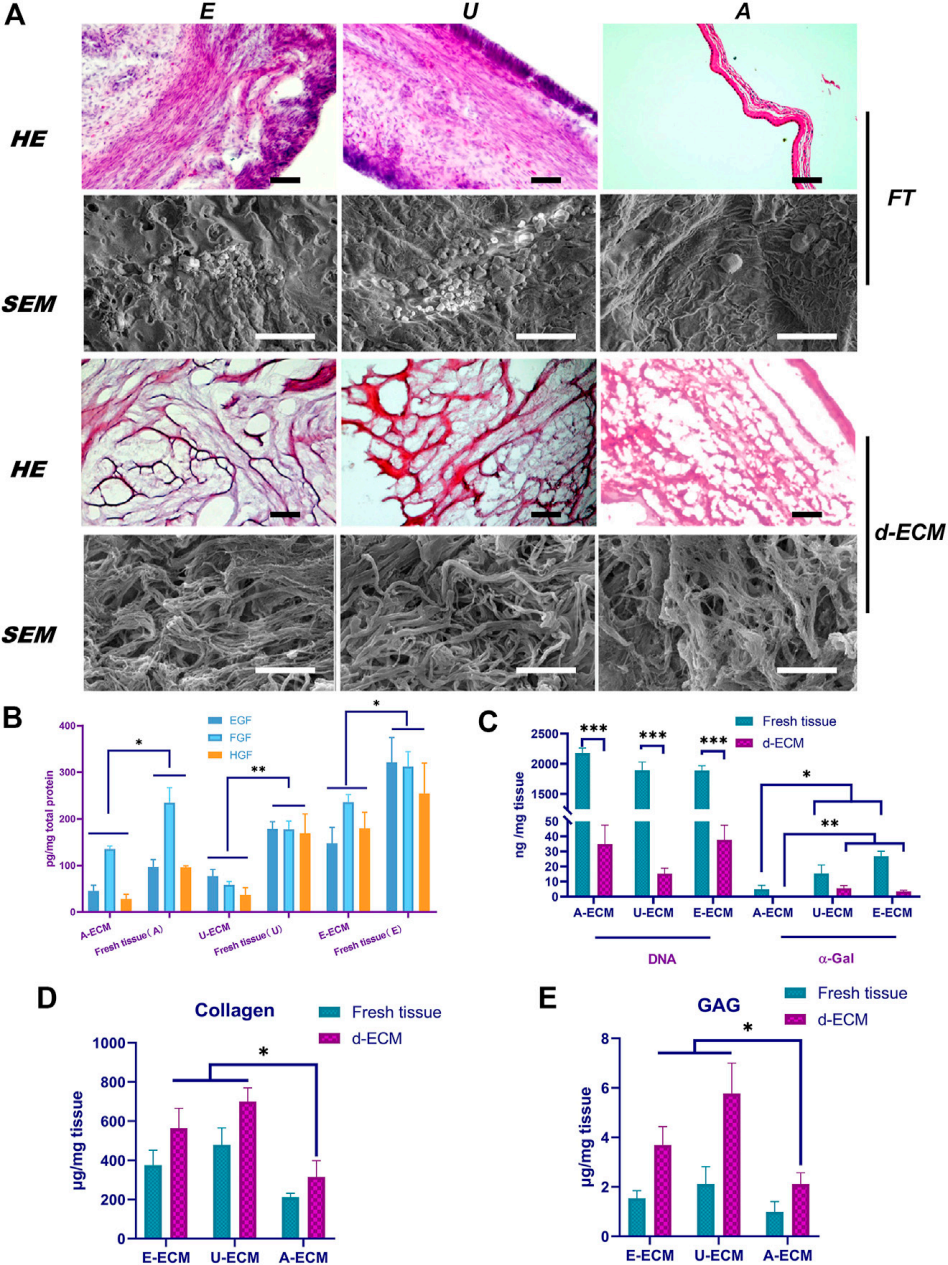
Comparison of the d-ECM and corresponding raw tissue microstructures and compositions. **(A)** Evaluation of the decellularization effects and microstructural characteristics of the ECMs via HE staining and SEM. **(B)** Quantitative evaluation of residual active factors in the three ECMs. **(C)** After decellularization, the amounts of residual DNA and α-gal were compared among the three groups and in fresh tissues. **(D,E)** The protection of ECM components in tissues after decellularization. *p < 0.05, **p < 0.01, and ***p < 0.001, one-way ANOVA with Tukey’s post hoc test. Bar = 400 μm (Top) and 50 μm (low) in the HE images and bar = 5 μm in the SEM image. FT, fresh tissue; A, amniotic membrane; E, endometrium-; U, urinary.

The structural morphologies of the samples were evaluated by HE staining. After decellularizing the samples under optimal conditions, we observed nearly no residual cellular components in the three types of d-ECMs by HE staining (Figure 1A), indicating that the decellularization process removed cells and cell fragments without obviously changing the morphologies or distributions of the ECMs. Assessment of the overall morphologies revealed that the structures of dE-ECM and dU-ECM were highly similar and looser than that of dA-ECM.

The structures of these three ECMs were similar under high magnification. The SEM results (Figure 1A) showed that the ECMs exhibited small pore-like structures on their surface composed of filaments. Compared with those of dA-ECM, the filaments of dE-ECM and dU-ECM were thicker, and the enclosed holes were larger. These results were consistent with the HE staining results.

### 3.2 Composition Detection

#### 3.2.1 Analysis of Residual Active Factors

The ECM contains abundant amounts of active factors, such as EGF, HGF, and bFGF. The ELISA results (Figure 1B) showed that these growth factors were still abundant even after the decellularization process. EGF, HGF, and bFGF (147.77 ± 34.13 pg/mg, 235.71 ± 16.65 pg/mg, and 179.57 ± 34.66 pg/mg, respectively) were most abundant in dE-ECM, whereas their levels were significantly reduced in dA-ECM (45.75 ± 12.14 pg/mg, 135.57 ± 6.16 pg/mg, and 28.25 ± 9.91 pg/mg) and dU-ECM (77.17 ± 14.41 pg/mg, 58.49 ± 7.01 pg/mg, and 37.33 ± 15.17 pg/mg) (*p* < 0.05). In general, the d-ECMs contained the growth factors EGF, HGF, and bFGF in amounts that varied among tissue types.

#### 3.2.2 Quantitative Detection of Residual DNA and α-Gal

The effects of the decellularization techniques were further confirmed by quantifying the residual DNA and α-gal content. Quantitative analysis of residual DNA (Figure 1C) revealed significantly reduced levels in the decellularized tissues compared to native tissues (*p* < 0.001), indicating that the decellularization process significantly reduced the DNA content in the ECM to below 50 ng/mg. The DNA levels before treatment were 2178.35 ± 85.13 ng/mg, 1893.77 ± 135.56 ng/mg, and 1890.92 ± 79.78 ng/mg in dA-ECM, dU-ECM, and dE-ECM, respectively; after decellularization, the DNA levels were reduced to 34.86 ± 12.66 ng/mg, 15.24 ± 3.75 ng/mg, and 37.62 ± 9.81 ng/mg, respectively, indicating that approximately 99% of the DNA was removed. The α-gal levels in fresh tissues, dU-ECM (15.38 ± 5.76 ng/mg), and dE-ECM (26.91 ± 3.24 ng/mg) were all higher than that in dA-ECM (4.93 ± 2.71 ng/mg) (*p* < 0.05). After treatment, α-gal was almost undetectable in dA-ECM, whereas some residual α-gal was detected in dU-ECM and dE-ECM (5.65 ± 1.77 ng/mg and 3.46 ± 0.78 ng/mg, respectively).

#### 3.2.3 GAG and Collagen Contents

The ECM molecules GAGs and collagen are important for cell proliferation and differentiation and play essential roles in remodelling processes by binding growth factors. The ELISA results (Figures 1D,E) showed enrichment of sulfated GAGs and collagen in the d-ECM, indicating that this decellularization method did not cause ECM loss. The GAG (1.54 ± 0.31 μg/mg and 2.11 ± 0.71 μg/mg) and collagen (564 ± 102 μg/mg and 701 ± 69 μg/mg) concentrations in dE-ECM and dU-ECM groups were significantly higher than those in dA-ECM (0.98 ± 0.43 μg/mg collagen and 315 ± 84 μg/mg GAGs) (*p* < 0.05). The compositional differences in GAGs and collagen in the decellularized and fresh tissues were attributed to the differences in their origin.

### 3.3 Effects on the Proliferation and Secretion Functions of Stromal Cells

**FIGURE 2:**
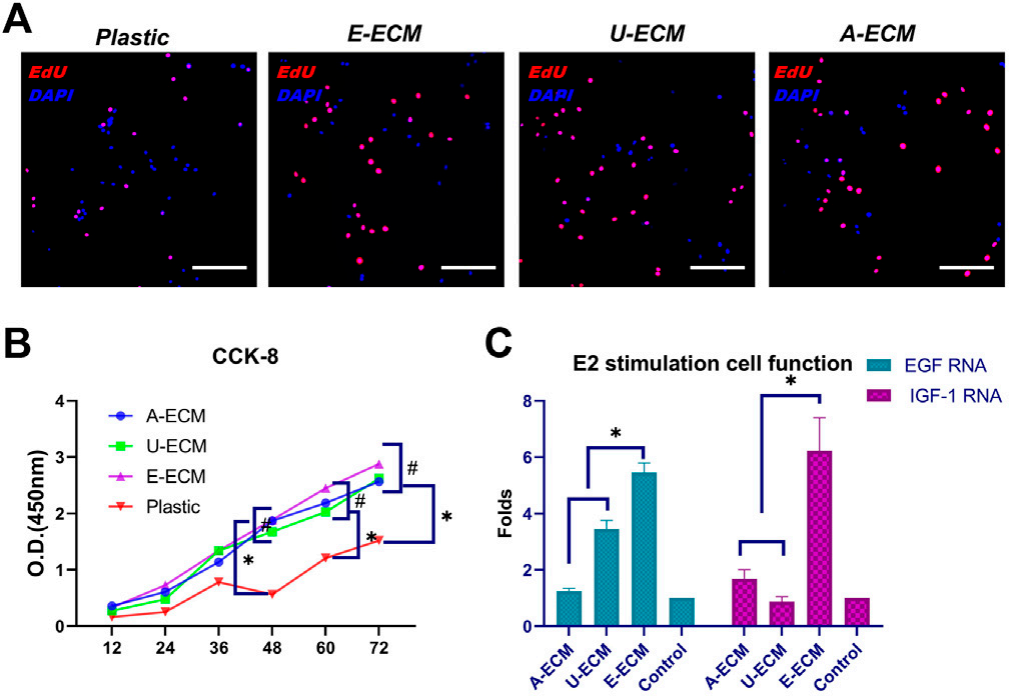
Measurement of cell proliferation and secretion functions after culture with three different substrates. The cell proliferation and quantitation results for stromal cells cultured with different substrate coatings **(A,B)** and evaluation of the secretion functions of stromal cells **(C)** after oestrogen stimulation under the above conditions. *p < 0.05 and ^#^ *p* > 0.05, one-way ANOVA with Tukey’s post hoc test. Bar = 50 μm.

The ECM provides mechanical and biochemical support and adhesion sites, which may increase the proliferation rate of cultured cells *in vitro*. 5′-Bromo-2′-deoxyuridine (EdU) and Cell Counting Kit-8 (CCK-8) are commonly used to reflect cell proliferation. Using the three ECMs as coating substrates, stromal cells were cultured for 12, 24, 36, 48, 60, and 72 h, and their proliferation rates were higher than that of cells cultured normally (Figures 2A,B, *p* < 0.05), suggesting that the d-ECMs played a role in promoting cell proliferation. However, at these time points, no significant differences were observed among the three ECMs (*p* > 0.05). VEGF and IGF-1 are mainly distributed in the uterine cavity of the epithelium, glandular epithelium, and stromal cells and periodical undergo changes. Physiologically, these proteins to stimulate hyperplasia of the endometrial glandular epithelium and stroma, and their synthesis is regulated by oestrogen. The oestrogen stimulation experiment results (Figure 2C) showed that the mRNA levels of both VEGF and IGF-1 were increased in the three exposed groups compared to the control group (plastic group; no substrate coating). The levels of both cytokines were significantly increased in dE-ECM (5.46 ± 0.34-fold for VEGF and 6.23 ± 1.17-fold for IGF-1). These results indicated that, under different ECM-coating culture conditions and stimulation with the same concentration of E2, stromal cells in the dE-ECM group were more sensitive to E2 stimulation and that the increase was significantly higher than that in the dA-ECM group (1.24 ± 0.11-fold for VEGF and 1.69 ± 0.32-fold for IGF-1) and dU-ECM group (3.45 ± 0.31-fold for VEGF and 0.87 ± 0.18-fold for IGF-1) (*p* < 0.05). Although the levels in the dA-ECM and dU-ECM groups were higher those in the control group, it was difficult to determine whether the effects of the d-ECMs on cell proliferation were actually different.

### 3.4 Inflammatory Response

**FIGURE 3:**
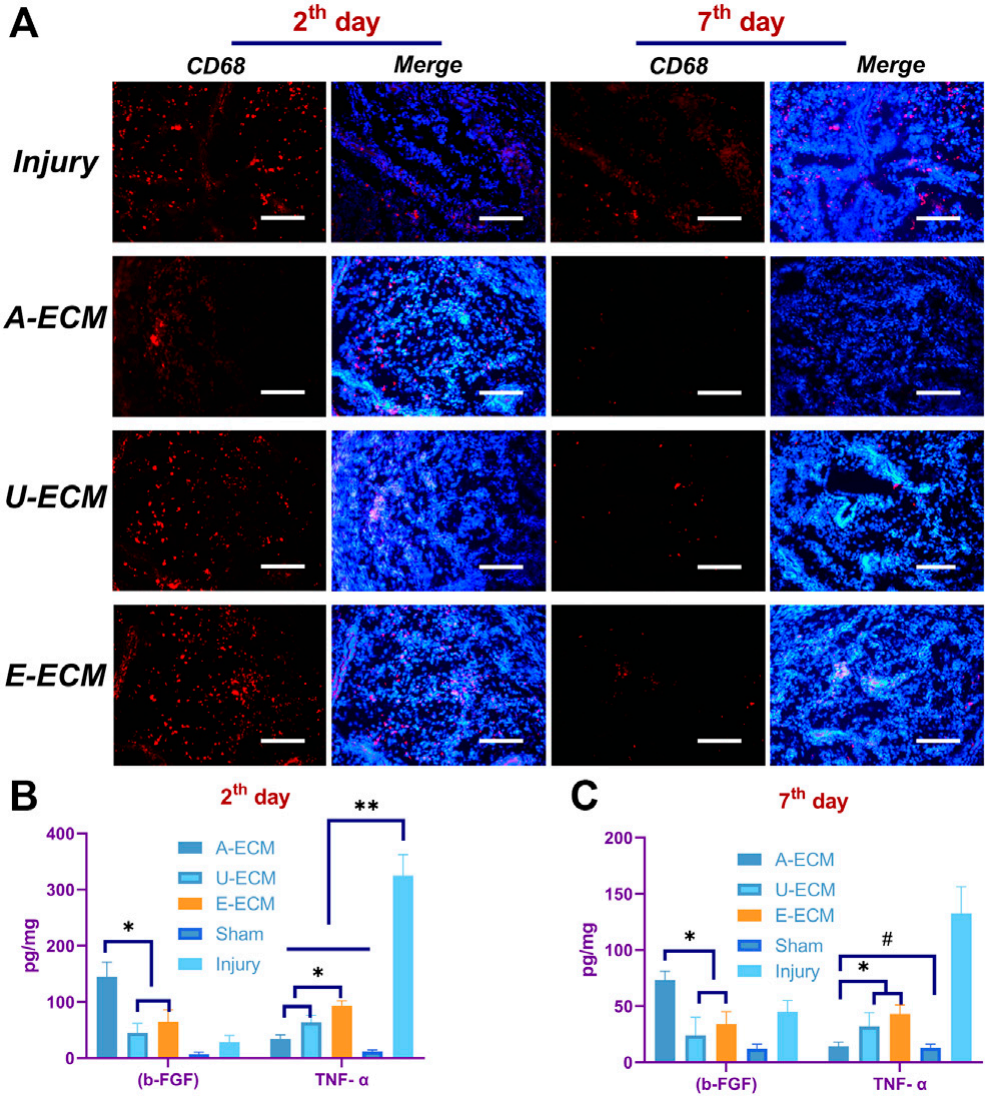
Local and systemic inflammatory conditions in the body after d-ECM implantation. **(A)** Local CD68 expression (macrophage indicator) at postoperative day 2 and 7 in all groups and quantitative comparison of the proinflammatory cytokine and anti-inflammatory cytokine levels at the corresponding time points **(B,C)**.*p < 0.05, **p < 0.01, and ^#^ *p* > 0.05, one-way ANOVA with Tukey’s post hoc test. Bar = 50 μm.

CD68 reflects the local inflammatory cell infiltration status. The results shown in Figure 3 A demonstrated that the lesion areas in each group were more substantially invaded by macrophages at postoperative day 2 due to various factors such as surgery, reflecting early local inflammatory responses. However, CD68 was expressed at a lower level in the dA-ECM group than in the other groups. At postoperative day 7, none of the lesion areas in the d-ECM implantation groups exhibited obvious CD68 ^+^ inflammatory cell infiltration, while numerous macrophages were still observed in the injury group. Again, these results indicate that the d-ECMs inhibited local inflammatory cells. In addition, the locally induced inflammatory response in the dA-ECM group was significantly less extensive than that in the dE-ECM and dU-ECM groups.

To evaluate possible inflammatory responses induced by the implantation of the three ECMs, the levels of inflammatory cytokines in local tissues were detected by ELISA. At day 2 after the operation, the inflammatory responses of the three experimental groups differed from those of the sham and untreated (injury) groups (Figure 3B). The anti-inflammatory cytokine b-FGF (144.54 ± 26.17 pg/mg) was expressed at a significantly higher level in the uterine tissues of the dA-ECM group than in those of the dE-ECM group (45.16 ± 16.79 pg/mg) and the dU-ECM group (64.57 ± 21.32 pg/mg), whereas the proinflammatory cytokine TNF-α (34.28 ± 7.30 pg/mg) was expressed at a lower level in the dA-ECM group than in the dE-ECM group (63.46 ± 12.55 pg/mg) and dU-ECM group (93.49 ± 8.18 pg/mg). These results suggest that the inflammatory response induced by dA-ECM was significantly less extensive than that induced by dE-ECM and dU-ECM.

At postoperative day 7 (Figure 3C), the levels of both b-FGF and TNF-α were significantly decreased in the d-ECM implantation groups compared to the injury group (45.13 ± 9.91 pg/mg for b-FGF and 132.59 ± 23.69 pg/mg for TNF-α). In particular, in the dA-ECM group, the level of the proinflammatory cytokine TNF-α (14.16 ± 4.33 pg/mg) was basically reduced to that in the sham group (13.07 ± 3.00 pg/mg for TNF-α) (*p* > 0.05). The above results show that the levels of the proinflammatory cytokine were significantly decreased and that those of the anti-inflammatory cytokines were significantly increased after d-ECM implantation compared to those in the injury group, indicating that the d-ECMs inhibited local inflammatory responses.

### 3.5 Graft Degradation and Antifibrotic Effect of d-ECMs

**FIGURE 4:**
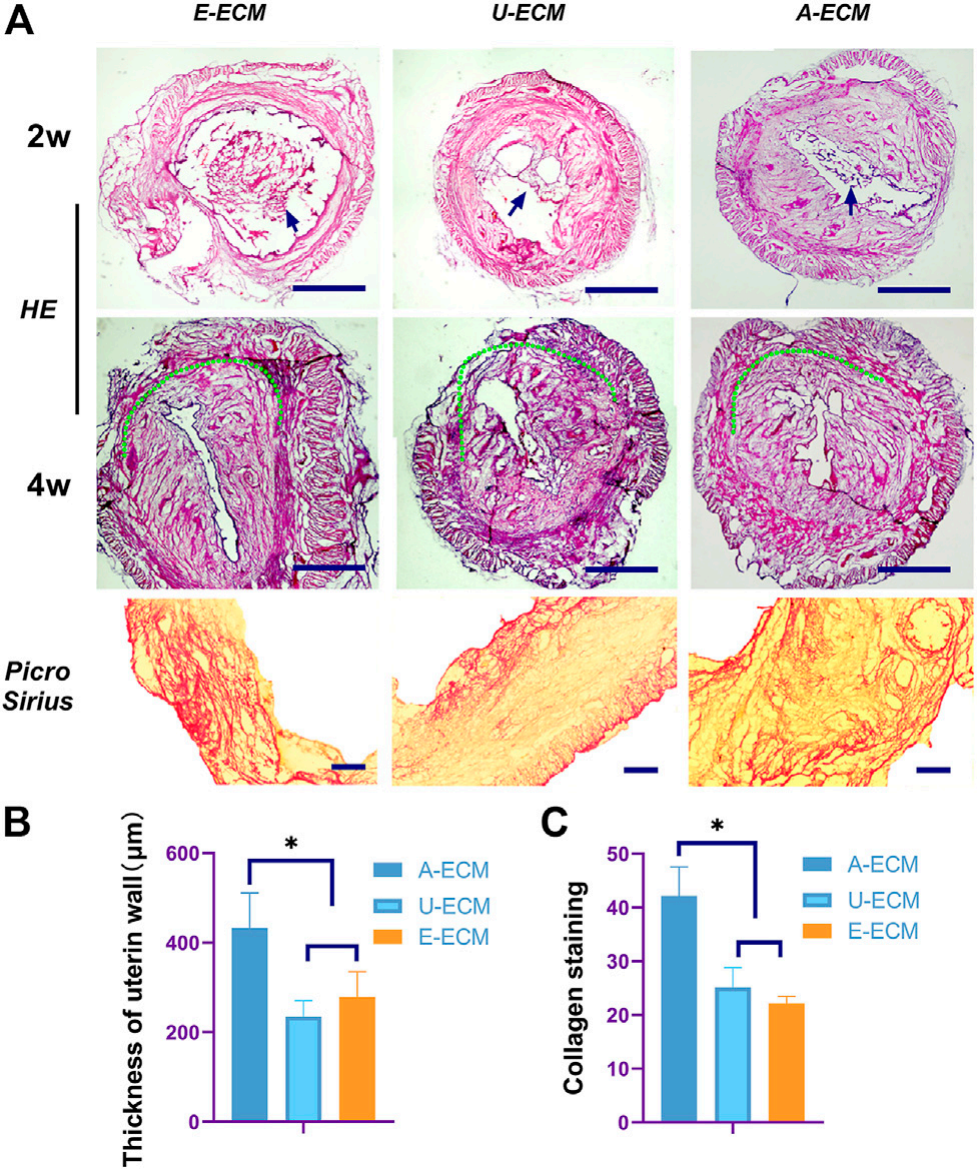
Morphological assessment of the regenerated endometrium. Two and 4 weeks after surgery **(A,B)**, HE and Picro Sirius staining were used to evaluate the thickness of the regenerated endometrium and the collagen contents **(C)** in all the groups.*p < 0.05, one-way ANOVA with Tukey’s post hoc test. Bar = 500 μm in the HE images and bar = 500 μm in the Picro Sirius staining images. Arrows shown the damaged area of the uterine horn in each group.

At 2 weeks after the operation, loose d-ECM debris was still observed in the uterine cavity. The d-ECM had been partially disintegrated (see Figure 4A, arrows), but it was obvious that many cells had infiltrated into the d-ECM. At 4 weeks after the operation, the graft in the uterine cavity was completely degraded, and no sign of the graft was observed in the damaged area.

**FIGURE 5:**
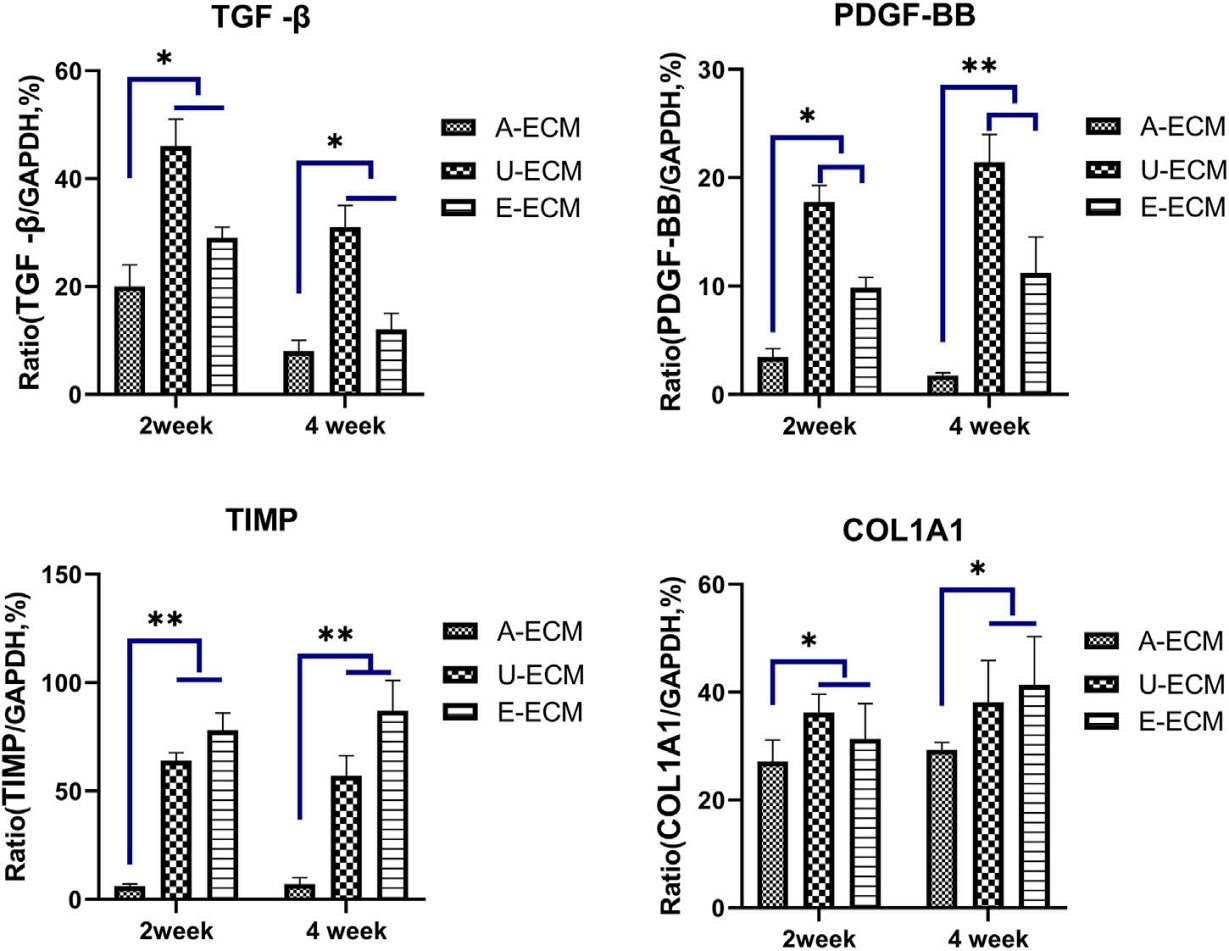
Postoperative mRNA levels of fibrosis-related cytokines in each group. At postoperative weeks 2 and 4, the mRNA expression levels of fibrosis-related cytokines (TGFβ, TIMP, PDGF-BB, and COL1A1) in the uterine tissues of the experimental groups were assayed by qRT-PCR and compared among the groups. *p < 0.05 and **p < 0.01, one-way ANOVA with Tukey’s post hoc test.

TGF-β, TIMPs, pPDGF-BB, and COLIA1 have been shown to be highly involved in the pathogenesis of IUA by promoting endometrial fibrosis. At postoperative week 2 and 4 (Figures 5A–D), the mRNA levels of profibrotic cytokines in uterine tissues of the dA-ECM group were downregulated compared to those in the dU-ECM and dE-ECM groups. Fibrosis was more obvious the in the regenerated endometria of the dU-ECM and dE-ECM groups, suggesting that dA-ECM implantation had a better long-term antifibrotic effect.

### 3.6 Revascularization and Cell Proliferation in the Regenerative Endometrium

**FIGURE 6:**
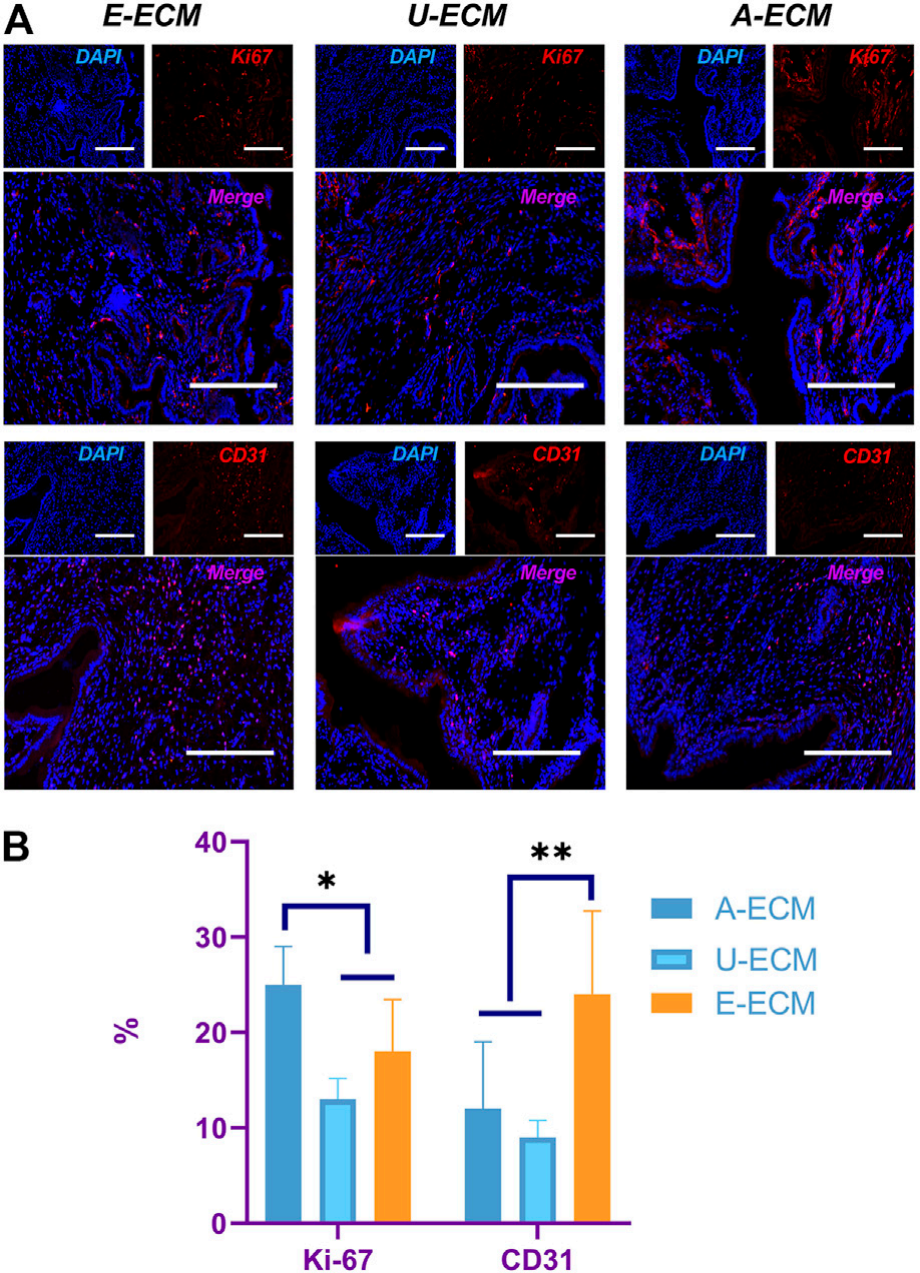
Evaluation of proliferative cells and vascular regeneration in the endometrium. At postoperative week 4, **(A)** Ki-67 and CD31 immunohistochemistry staining were used to compare cell proliferation and vascular endothelial cell regeneration in the endometrium, and the values were quantitated **(B)** for all the groups.*p < 0.05 and **p < 0.01, one-way ANOVA with Tukey’s post hoc test. Bar = 50 μm.

CD31 is a specific marker of vascular endothelial cells. Immunohistochemistry staining of CD31 (Figures 6A,B) was used to evaluate endometrial vascular regeneration after d-ECM implantation. At postoperative week 4, cells in three randomly selected fields (400×) were counted, revealing that CD31 (24 ± 8.74) expression was slightly higher in the dE-ECM group than in the dA-ECM (12.42 ± 7.24) and dU-ECM (9.68 ± 1.71) groups.

Ki-67 is used as an indicator of cell proliferation, and immunohistochemistry staining of Ki-67 (Figures 6A,B) was performed to assess the potential promotional effects of the d-ECMs on endometrial cell proliferation. At postoperative week 4, a number of proliferating cells were found in the regenerated endometria of the three groups. The number of proliferating cells in the dA-ECM group (25.53 ± 4.42) was significantly higher than that in the dU-ECM (13.15 ± 2.17) and dE-ECM (18.31 ± 5.44) groups.

### 3.7 Endometrial Regeneration

Endometrial tissue regeneration involves three important steps, regeneration of endometrial cells (including epithelial cells and stromal cells), regeneration of glands, and collage deposition.

**FIGURE 7:**
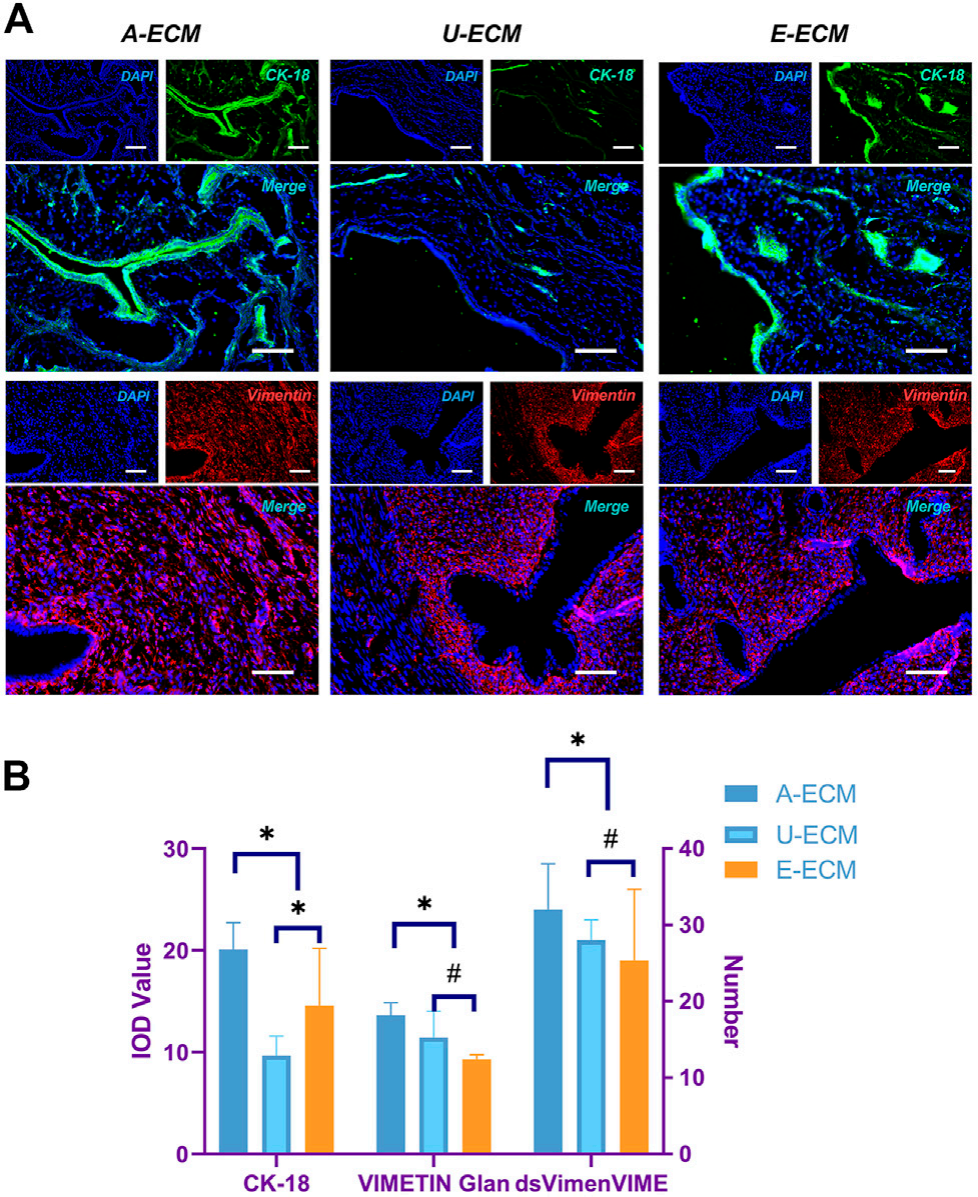
Protein expression of cytokeratin 18 (CK18) and vimentin in regenerative endometrial tissues. At postoperative week 4 **(A,B)**, CK18 and vimentin immunohistochemistry analyses were used to compare the regeneration of endometrial epithelial cells and stromal cells among all the groups. *p < 0.05 and ^#^ *p* > 0.05, one-way ANOVA with Tukey’s post hoc test. Bar = 50 μm.

At 2 and 4 weeks after the surgeries, HE staining (Figures 4A,B) showed that the injured uterine of the dA-ECM group (432.36 ± 78.14) had a thicker regenerative endometrial layer than that in the dU-ECM (235.18 ± 35.61 μm) and dE-ECM (278.47 ± 56.53 μm) groups. Immunofluorescence staining (Figures 7A,B) at postoperative week 4 showed that the expression levels of epithelial cell (CK18 26.78 ± 3.55) and stromal cell (vimentin 18.14 ± 1.66) markers and gland regeneration were higher (32 ± 6) in the dA-ECM group than in the dU-ECM and dE-ECM groups. Furthermore, regarding epithelial cell regeneration, the epithelial basement membrane (EBM) (14.56 ± 5.65) was regenerated better than the urinary bladder matrix (UBM) (9.31 ± 0.44). For stromal cells (vimentin) and gland regeneration, no significant differences were observed between these two groups. Picrosirius staining (Figures 4A,C) was used to evaluate collagen deposition by endometrial stromal cells, revealing that collagen production was significantly higher in the dA-ECM group than in the other groups.

### 3.8 Pregnancy Status

**FIGURE 8:**
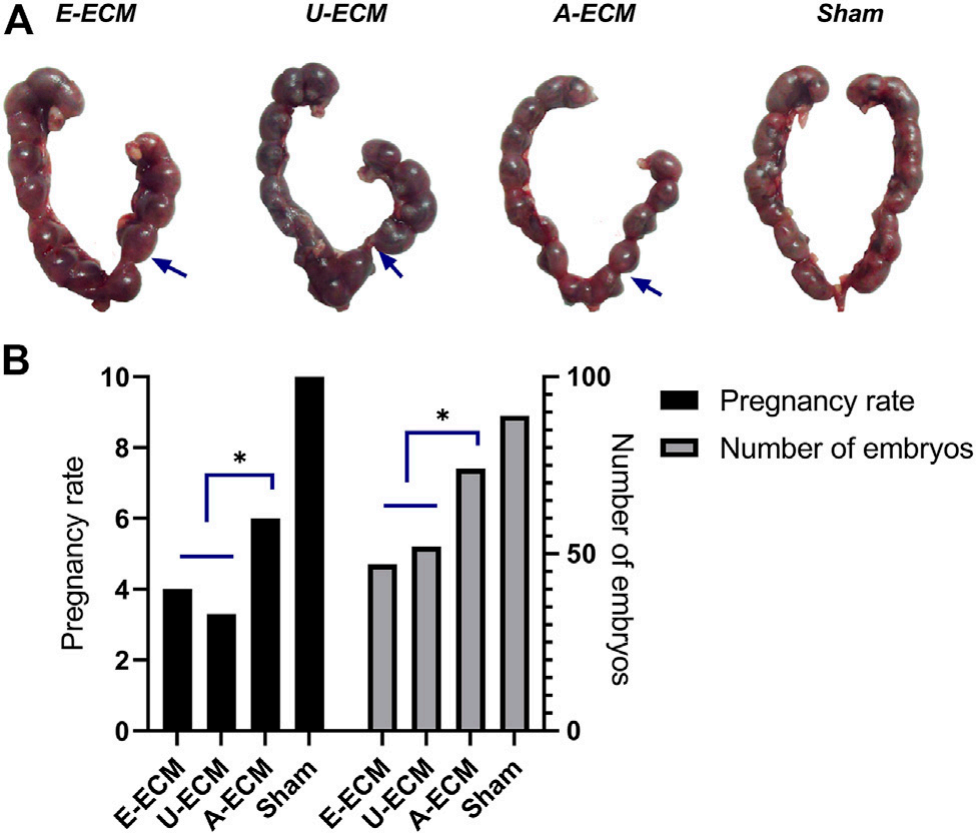
**(A,B)** Embryo breeding status of the injured uterus (blue arrows) in all groups.*p < 0.05, one-way ANOVA with Tukey’s post hoc test.Arrows shown the damaged area of the uterine horn in each group.

We compared the gestational statuses on the damaged sides of the uteri from different groups of animals (Figures 8A,B). After the female rats were caged with the male rats, the pregnancy rate in the sham-operated group was 100% (15/15), with an average of 8.9 embryos on the damaged side of the uterus. The pregnancy rate and mean number of embryos were 60% (9/6) and 7.40 in the dA-ECM group, 33% (5/10) and 4.70 in the dE-ECM group, and 40% (6/9) and 5.20 in the dU-ECM group, respectively. The damaged uterine horns of the animals in the dA-ECM group supported pregnancy (see arrows in Figure 8), but the number of embryos was significantly lower than that in the sham-operated group. Although the injured uterine in the dE-ECM and dU-ECM groups could conceive at different pregnancy rates, these two groups had significantly fewer detectable embryos (*p* < 0.05, vs. dA-ECM).

## 4 Discussion

The decellularization method recommended in this study was successfully utilized to obtain a satisfactory d-ECM. The d-ECMs derived from three tissues had distinct compositions, microstructures, and biochemical characteristics (Gilbert et al., 2006). Furthermore, although these three d-ECMs showed no differential effects on stromal cell proliferation, the *in vitro* culture results demonstrated that the use of dE-ECM as the coating substrate promoted the sensitivity of stromal cells to hormone stimulation. These results indicated that the three ECMs exerted differential effects on cellular behaviours (Tien and Nelson, 2014), which in turn led to their having differential effects on endometrial regeneration *in vivo*. Moreover, comparison of the *in vivo* study results further highlighted differences in the repair mechanisms of these three ECM types. The promotion of endometrial repair by dA-ECM was mainly mediated by anti-inflammatory and antifibrotic activity, and dA-ECM inhibited the inflammatory response at the early stage to a greater extent than the other two types. This strong immune tolerance (Shakouri-Motlagh et al., 2017; Hortensius et al., 2016) might have played a positive role in promoting endometrial regeneration and may have been the intrinsic factor underlying the better regeneration effect of dA-ECM compared with the other d-ECMs. Although dE-ECM created an internal environment suitable for endometrial cells to exert their functions, it did not have an advantage over dU-ECM in promoting endometrial regeneration at the early stage.

The native ECM is a three-dimensional structure containing a variety of extracellular molecules and is primarily composed of polysaccharides and proteins, including collagen and proteoglycans (Yao et al., 2019; Badylak et al., 2009). The amnion ECM is derived from the outer layer of foetal membranes and is the strength-bearing component, with high collagen and GAG concentrations (van der Linden et al., 1998); this ECM is crucial for the proposed maturation process in foetal membranes during the last week of pregnancy (Meinert et al., 2001). The UBM also has enhanced biomechanical properties and biological activities (Davis et al., 2018). Therefore, the GAG and collagen levels in the tissues of the dA-ECM and dU-ECM groups were higher than those in the dE-ECM group. Furthermore, these ECM components serve as the reservoirs of active factors (Yue, 2014). Studies have shown that d-ECMs contain a wide range of cytokines and growth factors (Boyd and Thomas, 2017). When applied to patients, active substances in the ECM are released into lesion sites to promote tissue regeneration. Generally, ECM–cytokine–cell interactions are thought to regulate endometrial cell proliferation, migration, and differentiation (Schonherr and Hausser, 2000). While the three ECMs did not influence endometrial stromal cell proliferation, dE-ECM clearly increased the sensitivity of endometrial stromal cells to E2, and we speculate that this response was associated with higher levels of residual active factors (such as VEGF, IGF-1, FGF, and HGF) in the dE-ECM group than in the other two groups. In addition, the structure and composition of dE-ECM most closely resemble the internal environment of the original tissues (Sainio and Jarvelainen, 2020); therefore, tissue-specific ECMs may play an important role in inducing stromal cells to exert their functions.

A prerequisite for d-ECM applications is to remove as many cellular and nuclear materials as possible to prevent the induction of bodily immune and inflammatory responses (Gilbert et al., 2006; Chakraborty et al., 2020). Previous studies have shown that the inflammatory response is an essential prerequisite for scarring and the most important pathological feature of uterine adhesions (Foix et al., 1966). Numerous studies have confirmed that residual ECM components, such as collagens, laminins, and polysaccharides, are highly conserved among species and tolerated well even by xenogeneic recipients (Gilbert et al., 2009; Badylak et al., 2009). Some scholars have defined three minimal criteria (Crapo et al., 2011; Chakraborty et al., 2020) that ensure effective decellularization. First, the product should contain less than 50 ng of dsDNA per mg of ECM (dry weight). Second, the DNA fragment should be less than 200 bp in length as determined by gel electrophoresis. Third, no nuclear material should be visible in the stained tissue sections. In the present study, the amounts of residual DNA in each of the d-ECM groups conformed to the above requirement, thereby preventing the recognition of allogeneic cellular antigens as foreign substances by the host, reducing the inflammatory response and preventing immune-mediated rejection of the tissue. Thus, the anti-inflammatory/antifibrotic activity of these three ECMs can be reasonably and scientifically evaluated. The *in vivo* results showed that the decellularized dA-ECM scaffold exhibited more suitable immune compatibility than the d-ECM scaffolds from other tissues, as evidenced by the decreased level of TGF-α and the increased level of bFGF, which plays a pathogenic role in IUA by promoting endometrial fibrosis (Foix et al., 1966). We speculate that this result was attributed to the decrease in the level of the α-Gal epitope in dA-ECM. The α-gal epitope is abundantly synthesized on glycolipids and glycoproteins in nonprimate mammals and New World monkeys by the glycosylation enzyme α-1,3 galactosyltransferase (α-1,3GT) (Huai et al., 2016). The α-gal epitope is the major antigen inducing complement-mediated cell lysis in the context of xenoimplantation (Wu et al., 2017). In fresh tissues herein, the α-gal epitope level in dA-ECM was significantly lower than that in the other two tissue types, which might explain why fresh AM has good immune compatibility. After the decellularization process, the α-gal epitope in the AM group almost completely disappeared, whereas a small amount was still observed in the other two groups. However, overemphasis on decellularization may damage the structure and components of the ECM (Gilpin and Yang, 2017). Overall, these results suggest that this decellularized matrix material with a low α-gal epitope level has very unique natural advantages when xenotransplanted into the human body.

Vascular regeneration has important significance for ameliorating endometrial functions, as it helps to maintain nutrient supplies and basic metabolism. However, more importantly, endometrial blood vessels form a vascular bed to undergo constant cycles of growth and regression during the reproductive life of females under the regulation of sex hormones (Rogers, 1996). Many studies have confirmed that ECM proteins can stimulate vascular regeneration (Honnegowda et al., 2015; Ma et al., 2020). The ECM plays a critical role in determining the proliferative, invasive and survival responses of local vascular cells to angiogenic growth factors (Saberianpour et al., 2018). Dynamic changes in both the ECM and local vascular cells coordinate angiogenesis regulation, and some studies have suggested that the ECM serves as a growth factor reservoir. Previous studies (Uttamsingh et al., 2008; Chen et al., 2020) have reported that TGF-β1 and EGF promote the proliferation and migration of various cell types and induce neovascularization, thereby playing a role in tissue repair. As a major angiogenesis induction factor, VEGF has been widely recognized. VEGF is not stored intracellularly but rather bound to the cell surface of the ECM (Ferrara, 2010). Two classes of VEGF binding sites have been identified in fibronectin. Furthermore, the proangiogenic activity of TGF-β1 has been confirmed and is strictly dependent on the composition and organization of the ECM (Neve et al., 2014). Herein, some active angiogenic substances remained detectable in the d-ECMs, with the highest amounts being observed in dE-ECM, which might explain its stronger promotional effect on vascular regeneration *in vivo*.

Endometrial tissue regeneration is the key for restoring the structure and function of the endometrium (Ji et al., 2020). Upon the assessment of stromal cell function, dE-ECM was shown to recapitulate and mimic the composition of the original tissue. Thus, dE-ECM may represent a promising approach for cell culture and embryo development in reproductive medicine that is more advantageous than nonspecific ECMs (like dU-ECM and dA-ECM in the current study) that most likely lack these characteristics. Therefore, we believe that dE-ECM, which most closely resembles the *in vivo* endometrial stromal environment, has the best promotional effect on endometrial regeneration. However, while almost no differences were observed between dE-ECM and dU-ECM, dA-ECM enhanced endometrial regeneration to a greater extent and was capable of supporting the implantation of a fertilized egg and maintaining a normal pregnancy. Thus, the roles of the tissue-specific ECMs were presumably not important for regeneration, and the proangiogenic active components of dE-ECM were also not the major influencing factors. Considering the anti-inflammatory and antifibrotic functions of dA-ECM at the early stage, we propose that these factors facilitate angiogenesis; prevent scar tissue from forming an early physical barrier that inhibits cell migration (Chen et al., 2019b); prevent immune inflammatory cells from secreting proinflammatory cytokines that induce an unfavourable internal environment for cell growth; and influence the survival, migration, and differentiation of recruited regenerative cells (especially stem cells) (Chakraborty et al., 2020). Therefore, we believe that anti-inflammatory and antifibrotics functions at the early stage are key to endometrial regeneration, which might explain why dA-ECM is more applicable, especially for skin burns and eye diseases (Arrizabalaga and Nollert, 2018; Shakouri-Motlagh et al., 2017).

## 5 Conclusion

The d-ECMs derived from three tissue types herein exhibited substantial structural and compositional differences, which may have played a decisive role in the biological characteristics of the cells. The implantation of d-ECM from AM (via alloimplantation/xenoimplantation) is safer and more reliable than the implantation of d-ECMs from other tissues. Although the ECMs derived from all three tissue types advantageously exerted antifibrotic effects, regulated the immune response, and promoted vascular regeneration after bodily implantation, dA-ECM exerted the best endometrial regeneration effect due to its anti-inflammatory and antifibrotic functions at the early stage. While dE-ECM is advantageously tissue-specific, it might not function as expected at the early stage of damage repair due to interference with inflammatory responses; at the late stage of repair, the d-ECM is rapidly degraded and thus cannot exert its positive effects as easily. Therefore, matching the unique advantages of d-ECMs with the specific requirements of the tissue repair process in time and space may be a focus of d-ECM design and application.

## Data Availability

The raw data supporting the conclusion of this article will be made available by the authors, without undue reservation.
